# Editorial: Micronutrients and Fatty Acids in Precision Nutrition Strategies

**DOI:** 10.3389/fnut.2021.788215

**Published:** 2021-12-09

**Authors:** Manja M. Zec, Irena Krga, Lizelle Zandberg, Cornelius M. Smuts

**Affiliations:** ^1^School of Nutritional Sciences and Wellness, University of Arizona, Tucson, AZ, United States; ^2^Centre of Research Excellence in Nutrition and Metabolism, Institute for Medical Research—National Institute of the Republic of Serbia, University of Belgrade, Belgrade, Serbia; ^3^Centre of Excellence for Nutrition, North-West University, Potchefstroom, South Africa

**Keywords:** precision nutrition, micronutrients, fatty acids, polyphenols, nutrigenetic biomarkers, microbiota

## Precision Nutrition

The importance of precision nutrition in health management strategies is gaining attention. Precision nutrition aims to translate individual molecular signatures into applicable personalized dietary and nutritional recommendations and ultimately a personalized dietary plan. Some endeavors have been made to customize macronutrient intake for individual predispositions, through the implementation of different dietary regimes, such as the Mediterranean diet with optimized fat intake (1), or a keto diet that favors fat intake (2). Huge interest, however, remains in supplying adequate amounts of essential microelements, individual nutrients, and bioactives, based on personal predispositions which include genetics, microbiota features, and molecular nutrition biomarker profiles. Having said that, this Research Topic reflects recent works dealing with nutrigenetic and gene *x* dietary/environment interactions, micronutrients, and microbiota responsiveness to bioactive supplementation, altogether evaluated in various health contexts.

## Microelements and Bioactives in Early Life Development and Healthy Aging

Early development largely depends on maternal nutritional competence, which primarily relies on intake but could also be modulated by endogenous factors such as the genetic background of a mother and even the developing fetus, both factors influencing the adequate nutritional supply of an offspring. The first 1,000 days concept requires appropriate delivery of essential nutrients, otherwise, a set of metabolic derangements may occur in malnourished children (3). In addition to the nutritional intake itself, a genetic make-up might modulate mother-offspring nutritional transfer. Thus, it has been suggested that a genetic variation in *SLC5A5* (rs775249401) gene locus might modulate transportation of iodine into breast milk, with A-allele carrier mothers being more efficient with iodine transportation to breast milk when exposed to inadequate iodine intake (Siro et al.). The results have been demonstrated in women of African descent and warrant more research in the race- and ethnic-specific context. On the other hand, a genome-wide association study across 7 million single nucleotide polymorphisms in Asian populations of Malay, Indian, and Chinese women, revealed that genetic variation (rs4588) in *GC* gene encoding for vitamin D binding protein was associated with low vitamin D status in mothers and cord blood, while the variation in *CYP2J2* (rs10789082) was associated with low vitamin D in the antenatal analysis only (Sampathkumar et al.). Precision nutrition tailored strategies based on adequate vitamin D supplementation during pre- and peri-conception periods could largely improve vitamin D deficiencies in both women and babies, potentially associated with immunological benefits and musculoskeletal health.

On the other side of the lifespan continuum, adequate supplementation of bioactives such as polyphenols and microelements from fruits and medicinal plants might improve healthy aging and prevent neurodegenerative disease through a panel of nutri(epi)genetic mechanisms, as well as gut microbiota-brain axis, as reviewed in Milošević et al.

## Polyphenols and Micronutrients in Immunity and Hemostasis

A body of research has recently been directed toward gut microbiota different organ axes, with gut microbiota eubiosis playing an essential role in immunological balance and prevention of a leaky gut through sustained intestinal integrity and low permeability. Fine modulation of gut microbiota profiles remains an outstanding goal for future personalized strategies, and a recent paper has shown that polyphenols from medicinal plants might have growth-stimulating effects on probiotic bacterial and yeast strains (Milutinović et al.).

The importance of microelements in immunological response has also been demonstrated in the burning topic of COVID-19 disease, with unprecedented effects on public health globally. In adult Serbian subjects, but not in a pediatric population, variations in genes involved in vitamin D metabolism (*DHCR7/NADSYN* rs12785878 and *CYP2R1* rs10741657 variants) were linked with severe COVID-19 disease (Kotur et al.), implicating the potential of vitamin D-based precision supplementation strategies in boosting immunological response toward more efficient COVID-19 prevention and suppression.

Precision nutrition supplementation strategies of iron might also depend on the genetic variation coding for fibrinogen and FXIII molecules, as indicated in the recent epidemiological cohort of black African descent (Rautenbach et al.). In this study, biomarkers of iron status, such as ferritin and transferrin, were shown to be linked with fibrinogen, fibrin formation, and fibrinogen lysis, implicating a role of circulating iron in hemostasis.

## Bioactives and Nutrients in Chronic Diseases

Due to the increased incidence of cardiovascular disease worldwide, associated with a striking increase in obesity, related to nutritional transitions toward Westernized dietary patterns, novel personalized approaches in cardiometabolic conditions are highly desirable.

Zinc is involved in the inflammatory response and disturbed zinc homeostasis is an early feature of cardiovascular disease. Zinc status is essential for optimal cellular and tissue proliferation, hormonal and immunological balance, and randomized controlled zinc interventions to alleviate cardiovascular disease, as well as nutrigenetic implications of zinc transporter proteins and metallothionein, are much needed (Knez and Glibetic). The necessity of zinc supplementation has also been established in reportedly zinc-deficient hemodialysis patients, who also presented with an inadequate long-chain omega-3 fatty acid intake (Takic et al.), indicating potential interaction between fatty acid and micronutrient metabolism. Furthermore, the interaction between dietary bioactives i.e., polyphenols from Aronia berry and polyunsaturated fatty acid metabolism, has also been reported in dyslipidemic women, with those consuming polyphenol-rich berry juice having a lower inflammatory arachidonic acid/eicosapentaenoic acid ratio (Stojković et al.). The same study demonstrated that habitual polyphenol intake also induced decreases in Long Interspersed Nucleotide Element-1 methylation levels in peripheral blood leukocytes, which was dependent on *MTHFR* variant allele (C677T), indicating nutri(epi)genetic forces elicited by polyphenol consumption. Aside from microelements and bioactives, fatty acid profiles are influenced by the quality of a diet. In obese subjects, *FADS2* rs174583 variant, involved in dietary fatty acid conversion, interacted with the level of adherence to either Dietary Approach to Stop Hypertension or Mediterranean diet, finally affecting triglyceride concentration/diastolic blood pressure in women/men and glucose serum levels, respectively (Khodarahmi et al.).

Finally, precision nutrition strategies are of special importance in populations with specific nutritional and dietary needs, which was demonstrated in a recent study in underground coal miners, where unexpected direct correlations between serum vitamin D levels and anthropometric and biochemical indicators were demonstrated, the latter commonly indicative of obesity, metabolic syndrome, and fatty liver disease (Šarac et al.).

## Foundation for Future Studies

In order to implement precision nutrition postulates in practice, further race-, ethnicity-, and population-specific randomized controlled studies are needed to validate responsiveness to a particular nutrient and dietary intervention depending on the holistic interpretation of individual genetic and molecular biomarker profiles. Further research should put additional attention on population groups with specific nutritional and dietary needs, such as occupational groups and athletes.

## Author Contributions

MMZ wrote the manuscript and IK, LZ, and CMS reviewed the manuscript. All authors approved the final version of the manuscript.

## Conflict of Interest

The authors declare that the research was conducted in the absence of any commercial or financial relationships that could be construed as a potential conflict of interest.

## Publisher's Note

All claims expressed in this article are solely those of the authors and do not necessarily represent those of their affiliated organizations, or those of the publisher, the editors and the reviewers. Any product that may be evaluated in this article, or claim that may be made by its manufacturer, is not guaranteed or endorsed by the publisher.
